# Phenotype Fingerprinting Suggests the Involvement of Single-Genotype Consortia in Degradation of Aromatic Compounds by *Rhodopseudomonas palustris*

**DOI:** 10.1371/journal.pone.0004615

**Published:** 2009-02-26

**Authors:** Tatiana V. Karpinets, Dale A. Pelletier, Chongle Pan, Edward C. Uberbacher, Galina V. Melnichenko, Robert L. Hettich, Nagiza F. Samatova

**Affiliations:** 1 Computational Biology Institute, Oak Ridge National Laboratory, Oak Ridge, Tennessee, United States of America; 2 Computer Science and Mathematics Division, Oak Ridge National Laboratory, Oak Ridge, Tennessee, United States of America; 3 Biosciences Division, Oak Ridge National Laboratory, Oak Ridge, Tennessee, United States of America; 4 Chemical Sciences Division, Oak Ridge National Laboratory, Oak Ridge, Tennessee, United States of America; 5 Department of Plant Sciences, University of Tennessee, Knoxville, Tennessee, United States of America; 6 Biosystems Engineering & Soil Science Department, University of Tennessee, Knoxville, Tennessee, United States of America; 7 Department of Computer Science, North Carolina State University, Raleigh, North Carolina, United States of America; Center for Genomic Regulation, Spain

## Abstract

Anaerobic degradation of complex organic compounds by microorganisms is crucial for development of innovative biotechnologies for bioethanol production and for efficient degradation of environmental pollutants. In natural environments, the degradation is usually accomplished by syntrophic consortia comprised of different bacterial species. This strategy allows consortium organisms to reduce efforts required for maintenance of the redox homeostasis at each syntrophic level. Cellular mechanisms that maintain the redox homeostasis during the degradation of aromatic compounds by one organism are not fully understood. Here we present a hypothesis that the metabolically versatile phototrophic bacterium *Rhodopseudomonas palustris* forms its own syntrophic consortia, when it grows anaerobically on *p*-coumarate or benzoate as a sole carbon source. We have revealed the consortia from large-scale measurements of mRNA and protein expressions under *p*-coumarate, benzoate and succinate degrading conditions using a novel computational approach referred as phenotype fingerprinting. In this approach, marker genes for known *R. palustris* phenotypes are employed to determine the relative expression levels of genes and proteins in aromatics versus non-aromatics degrading condition. Subpopulations of the consortia are inferred from the expression of phenotypes and known metabolic modes of the *R. palustris* growth. We find that *p*-coumarate degrading conditions may lead to at least three *R. palustris* subpopulations utilizing *p*-coumarate, benzoate, and CO_2_ and H_2_. Benzoate degrading conditions may also produce at least three subpopulations utilizing benzoate, CO_2_ and H_2_, and N_2_ and formate. Communication among syntrophs and inter-syntrophic dynamics in each consortium are indicated by up-regulation of transporters and genes involved in the curli formation and chemotaxis. The N_2_-fixing subpopulation in the benzoate degrading consortium has preferential activation of the vanadium nitrogenase over the molybdenum nitrogenase. This subpopulation in the consortium was confirmed in an independent experiment by consumption of dissolved nitrogen gas under the benzoate degrading conditions.

## Introduction

Understanding cellular processes underlying the anaerobic degradation of complex organic compounds by microorganisms is crucial for development of innovative biotechnologies for production of effective energy alternatives, such as bioethanol and hydrogen gas from lignocellulose and biological wastes [1], and for efficient removal of toxic compounds produced by petroleum spills and incomplete combustion of fossil fuels [2]. In natural environments, the anaerobic degradation of aromatic compounds is usually accomplished by microbial consortia characterized by synergetic nutritional interactions of the involved microorganisms [3], [4]. These interactions can provide the most efficient use of available sources of energy, respiration, carbon, and nutrients. Terminal electron acceptors for anaerobic respiration can be used in sequence: initiating from the most favorable acceptors, such as nitrates, with the greatest relative energy yield and proceeding to iron, sulfate and methanogenesis [5]. This strategy reduces efforts required for maintenance of the redox homeostasis at each syntrophic level.

Anaerobic degradation of aromatic compounds with ring substituents by a single organism also proceeds through initial utilization of the substituent and transient accumulation of intermediates, which are more resistant to degradation [6]–[8]. *R. palustris*, a model organism for studies of anaerobic degradation of aromatic compounds, can readily degrade side chains of phenylalkanoates, such as cinnamate or coumarate (a hydroxyl derivative of cinnamic acid), yielding acetyl groups for biosynthesis or for energy production. Benzoate or 4-hydroxybenzoate is initially released by the organism as energetically more difficult to degrade [7], [8], but completely degraded lately. *R. palustris* implements all key steps of aromatics degradation including ring reduction, ring cleavage, and β-oxidation on its own [9], [10]. Cellular mechanisms that maintain the redox homeostasis of *R. palustris* during this complex set of biochemical reactions are not fully understood. Anaerobic oxidation of benzoate depends on availability of terminal electron acceptors, and the bacterium must have mechanisms to balance the activity of pathways that generate and consume reductants. One such mechanism may be the extreme metabolic versatility of *R. palustris* growing by anoxygenic photosynthesis, aerobic or anaerobic respiration and fermentation, fixation of nitrogen gas, or utilization of carbon through CO_2_ reduction using H_2_ as an electron donor [11], [12]. This set of phenotypes is similar to phenotypes found in natural multi-microbial consortia degrading aromatic compounds anaerobically [3], . The phenotypes represent different modes of the bacterial growth and occur in different bacterial organisms. They don't coincide in one growing cell. We can speculate that *R. palustris* may employ these phenotypes through differentiation in several cellular subpopulations with different metabolic modes of the growth. The formation of such single-genotype consortium would be an efficient way to decrease efforts for maintenance of the redox homeostasis in each individual subpopulation during aromatic compound oxidation.

The idea that genetically identical but phenotypically different cell subpopulations of one bacterial organism may be involved in degradation of an aromatic compound through syntrophic relationship with each other is novel and very challenging for validation. Even a discovery of this biological phenomenon is difficult. The composition of multi-microbial consortia is usually identified by culture-based isolation of the individual members of the consortia or by sequence-based molecular phylogenetic analysis [3]. These approaches, however, are hard to apply when subpopulations in the consortia are represented by the same organism. In this study we propose to reveal the consortia from the large-scale transcriptomics and proteomics experiments that give a global overview of mRNA and protein expressions during the degradation of the aromatic compounds by *R. palustris*. We have compared the expressions with those observed during the utilization of a non-aromatic carbon source (succinate) and calculated average changes in a set of marker genes/proteins represented phenotypes that are specific to distinct subpopulations of by *R. palustris*. Statistical significance of each phenotype expression was estimated by comparing changes in the marker genes with a randomly sampled gene set of the same size using t-statistics. The phenotypes expressed significantly in the condition of aromatic compound degradation were attributed to distinct subpopulations of *R. Palustris* considering its known modes of the growth and thermodynamic and energetic plausibility of the phenotypes co-activation in one cell. Then we analyzed the expressed/suppressed genes, pathways and predicted operons to provide additional evidence for the proposed consortium subpopulations. Finally, we reproduced *R. palustris* culture growth under condition of benzoate utilization to confirm the presence of the nitrogen fixing subpopulation in this condition by measuring changes of total nitrogen and ammonium in the culture.

## Results

### Hypothesis statement

The involvement of phenotypically different but genetically identical subpopulations in the degradation of aromatic compounds stems from known thermodynamic constrains on co-activation in one cell of some phenotypes that represent known metabolic modes of the *R. palustris* growth.

In bacteria the consumption of alternative sources of nutrition and respiration is usually suppressed by multiple regulatory mechanisms [3], [17]–[20]. An individual bacterial cell takes up and utilizes only one carbon source, one nitrogen source and one terminal electron acceptor at a time and leaves the other substrates available in the medium for later use. A combination of sources of energy and respiration utilizing by a bacterium in a particular environment characterizes the mode of the bacterium metabolism. Each mode is evolved to optimise the use of energy for utilization of the available electron donor/acceptor, nitrogen source and carbon source. Although *R. palustris* has extraordinary metabolic versatility, it usually grows anaerobically by one of the two major modes of metabolism: photoautotrophic (energy from light and carbon from carbon dioxide) or photoheterotrophic (energy from light and carbon from organic compounds) [21]. Similar to other hydrogen producing photosynthetic bacteria, *R. palustris* cannot combine the N_2_ fixation with the degradation of complex organic compounds or with the assimilation of CO_2_ [22]–[24]. Typical carbon sources and electron donors for *R. palustris* utilizing nitrogen gas at the expense of solar energy are small-chain organic acids, like lactate, malate, and formate. Switching from one metabolic growth to another requires transcriptional reprogramming of the cell. The cell stops proliferation and goes into a stationary phase, in which no increase in the number of cells is observed. After reprogramming the cell renews the exponential growth.

As far as *R. palustris* can completely degrade benzoate when it is growing with ammonium sulphate as the only source of fixed nitrogen, it was suggested that the organism has this specific mode of growth (Table 1., Eq. (1) and (2)). However, the complete oxidation of benzoate produces 30 electrons and requires terminal electron acceptors, which are not specified for this mode of growth. *R. Palustris* has a dissimilatory nitrite reductase [25] and can utilize nitrates as terminal electron acceptors, but the dissimilitary sufate reductase is absent from the genome of the organism. The genome has only enzymes involved in the assimilatory sulfate reduction to sulfite through acceptance of 2 electrons (Eq. (2) in Table 1), namely, three consecutive genes CysN, CysD, and CysH encode putative ATP-sulfurylase large subunit, sulfurylase small subunit, and putative phosphoadenosine phosphosulfate reductase. The *R. Palustris* has, however, more efficient electron sinks for balancing the oxidation reactions through reduction of nitrogen gas and carbon dioxide (Eqn. (8) and (10) in Table 1). But these processes would require different metabolic modes of the *R. palustris* growth.

**Table 1:** Basic components of the putative electron donor and electron acceptor reactions under different modes of the R. palustris growth (the reactions are written according to Zwolinski et al. [3] ).

Mode of growth	Electron acceptor reactions		Potential electron donor reactions	
**Homogeneous cell population**				
Benzoate degradation	Benzoate oxidation to CO_2_	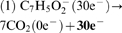	Sulfate reduction to sulfite and nitrate reduction to nitrite	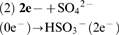 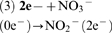 (nitrates were not supplied)
**Consortia**				
Benzoate degradation	Benzoate oxidation to acetate	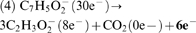	Not determined	Not determined
Acetate utilization	Acetate oxidation to formate	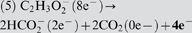	Not determined	Not determined
Nitrogen gas fixation	Formate oxidation to CO_2_	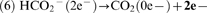	Nitrogen fixation	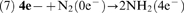
CO2 assimilation	H_2_ oxidation to protons	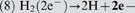	CO2 reduction to methane	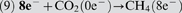

We propose that *R. palustris* balances oxidation-reduction reactions through differentiation into subpopulations implementing different modes of growth. A subpopulation consuming an aromatic compound, like benzoate, doesn't oxidize it completely to CO_2_ but rather produces an intermediate, like acetate or formate, and releases the intermediate into the environment (Table 1, Eqn. (4) and (5)). The incomplete oxidation decreases the requirement of terminal electron acceptors and energy in the cell. In addition, the released low chain carbons and hydrogen provide carbon sources and electron donors for utilization by *R. palustris* subpopulations implementing distinct modes of growth through nitrogen gas fixation and carbon dioxide assimilation. Thus, the differentiation of one genotype into phenotypically distinct subpopulations provides a mechanism to invoke efficient electron donors for complete degradation of benzoate.

### Hypothesis testing

To validate the hypotheses we have compared the *R. palustris* growth on aromatic compounds (benzoate and *p*-coumarate) versus the growth on non-aromatic compound succinate by calculating log_2_ ratio of gene and protein expressions from a large-scale microarray and proteomics study of the organism [26]. The main differences between the oxidation of benzoate vs succinate is that the former requires a set of specific enzymes involved in the aromatic ring degradation and a significant electron sink. According to the hypothesis, the shortage of electron acceptors should lead to differentiation of *R. palustris* cells under benzoate degrading conditions into subpopulations expressing distinct phenotypes. The activated phenotypes may include benzoate utilization, carbon dioxide utilization, formate utilization, nitrogen-fixation, and hydrogen gas utilization. Each of these phenotypes is characterized by a set of activated genes. As an example, genes representing structural components and accessory proteins of nitrogenases, enzymes involved in nitrogen gas fixation, may be considered as marker genes of nitrogen fixing *R. palustris* phenotype [11], [27]. Genes encoding enzymes of the Calvin cycle, represented in the *R. palustris* genome by *cbb_I_* and *cbb_II_ gene* clusters [28] may be considered as marker genes of CO_2_ utilizing phenotype. We can expect, therefore, that, the genes/proteins represented the phenotypes must be up-regulated, if we consider, for example, the log ratio of the gene or protein expressions in the benzoate degrading condition vs succinate degrading condition On the other hand, genes/proteins representing processes those are common for both conditions, like ribosome synthesis or photosynthesis, or the random population diversity will not show significant difference in the expression; enzymes involved in the utilization of succinate will be, likely, downregulated under benzoate versus succinate degradation.

Thus, our null hypothesis for each tested phenotype was that benzoate degrading condition doesn't show a significant difference vs succinate degrading condition in the average expression of genes/proteins representing the phenotype. The alternative hypothesis was that benzoate degradation leads to activation of genes/proteins representing the phenotypes that are characteristics of the specific modes of the *R. palustris* growth and, therefore, of distinct subpopulations of the organism. The set of genes (although refer as marker genes/proteins) specific for a phenotype may be considered as its fingerprint, and the average relative expression of the marker genes/proteins in the benzoate versus succinate degrading condition may characterize the phenotype expression under the benzoate degradation. Similar assumption can be made for *p*-coumarate degrading condition when it is compared with succinate degradation.

We have employed t-statistics to test the hypothesis. Because the distribution of log_2_ ratios of the gene and protein expressions in the considered datasets were close to normal (Figure S1), we preferred to use the Student's t-test instead of a non-parametric statistical hypothesis to provide a qualitative interpretation of the test results in terms of the phenotype expression. To assess the truth of the null hypothesis we have compared the mean expression of the marker genes representing each phenotype proposed by the hypothesis (test sample) with the mean expression of randomly sampled similar-size cluster of genes (random or permuted sample) by comparison of the means using standard deviations of the samples (see Material and Methods for details). Permuting the tested dataset for the comparison we simulated gene/protein expressions in a homogenous cell population and compared it with the expression of specific phenotypes representing subpopulations. We refer to this statistical analysis as phenotype fingerprinting. A novel aspect of the method is the consideration of each marker gene as a replicate to increase the number of replicates for presentation of each phenotype in the statistical analysis. This increase allowed a reliable detection (p<0.05) of low-expressed phenotypes representing a non-dominating subpopulation in the consortia, when the expression of some individual marker genes might be not significant because of a low size of the subpopulation.

Results of the analysis revealed phenotypes that are activated by *R. palustris* in benzoate/*p*-coumarate degrading conditions and, therefore, the potential metabolic mode of growth implemented by each cell subpopulation. Although the statistical test itself doesn't discriminate the subpopulations, the thermodynamic constrains on phenotype coexistence in one cell and known modes of *R. palustris* growth allowed us to infer the subpopulations.

### Fingerprinting of *R. palustris* phenotypes using marker gene

**Figure 1:**
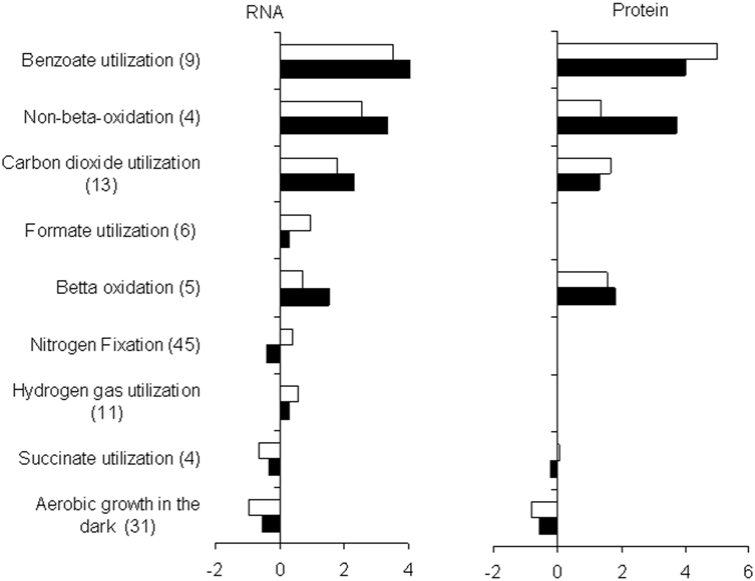
Expression of R. palustris phenotypes under p-coumarate (black columns) and benzoate (white columns) degrading conditions if compared with growth on succinate. The expression is calculated as average log2-ratios of the marker genes representing the phenotype (See Table S1 for the list of genes). The number of marker genes in each cluster is given in parenthesis. Calculated *p*-values for phenotype expressions and individual expressions of marker genes and proteins are given in the Table S2 and are discussed in the text. Some phenotypes are not revealed at the protein level, because their markers genes are represented by membrane proteins, like hydrogenase, nitrogenase, or formate hydrogenlyase. Detection of membrane proteins by LC-MS/MC is more difficult [41]–[43].

Known marker genes of nine *R. palustris* phenotypes (Table S1) were obtained from results of previous biological studies of the bacterium [11], [12], [27]–[31]. The phenotypes included: benzoate utilization, *beta*-oxidation, non-*beta*-oxidation, carbon dioxide utilization, formate utilization, nitrogen-fixation, hydrogen gas utilization, aerobic growth in the dark, and succinate utilization. Expressions of the phenotypes or their fingerprints at the level of mRNAs and proteins are presented in Figure 1 and Table S2. Most studied phenotypes, except utilization of succinate, showed statistically significant up- or down-regulation (*p*<0.05) under aromatic compound degrading growth conditions versus succinate utilizing growth condition. The activity of most phenotypes is confirmed at the level of genes and proteins. Relative changes in the expression of the phenotypes, however, varied from 2-fold decrease (for aerobic growth in the dark) to 10-fold increase (for benzoate utilization) indicating essential differences in the number of bacterial cells implementing the phenotypes in each growth condition. Anaerobic degradation of *p*-coumarate and benzoate, compared to growth on succinate, is accompanied by decrease in the abundance of mRNA and protein markers for two *R. palustris* phenotypes: aerobic growth in the dark and the utilization of succinate as a carbon source. At the same time, mRNAs and proteins related to utilization of benzoate (CoA ligation, ring reduction, and ring cleavage) show from 7 to 10-fold increase in the abundance (Figure 1). Proteins and mRNAs related to *β*-oxidation and non- *β*-oxidation also show up-regulation under *p*-coumarate and under benzoate degrading conditions. Both routes of oxidation, however, have consistently greater up-regulation at the level of genes and proteins under *p*-coumarate degrading condition.

Although benzoate utilization phenotype and *β*- and non-*β*-oxidation routes for conversion of *p*-coumarate to 4-hydroxybenzoate are obviously dominating when *R. palustris* degrades the aromatic compounds, CO_2_ utilization phenotype and hydrogen gas utilization phenotype are also present in both degrading conditions (Figure 1). The expression of the latter phenotypes is 2–8 times less than the expression of benzoate utilization phenotype and β-oxidation phenotype, but statistically significant (*p*<0.02−0.0001) at the level of marker genes and identified marker proteins.

Surprisingly, the benzoate degrading condition is also characterized by statistically significant activation of nitrogen fixation phenotype (*p* = 0,000053) and by formate utilization phenotype (*p* = 0.036). These phenotypes are absent, when *R. palustris* is growing on *p*-coumarate or succinate (Figure 1). High statistical significance of the nitrogen fixation phenotype expression is explained by relatively low variability in up-regulation of 45 genes representing this phenotype (Table S2).

In conclusion, the phenotype-based screening of gene expression and protein abundance under *p*-coumarate and benzoate degrading conditions versus succinate utilization condition shows that *p*-coumarate degrading condition is characterized by presence of 5 phenotypes: benzoate utilization, β-oxidation, non-β-oxidation, CO_2_ utilization, and H_2_ utilization. Benzoate degrading conditions are also characterized by presence of nitrogen fixation and formate utilization phenotypes in addition to phenotypes listed for *p*-coumarate degrading condition.

### Attribution of the phenotypes to *R. palustris* subpopulations in the consortia

Considering major modes of metabolism of *R. palustris* reviewed above, and co-existence different phenotypes in one microbial cell, the expressed phenotypes are attributed to different cellular subpopulations of the bacterium. We propose that the dominating subpopulation of *R. palustris* utilizes benzoate or *p*-coumarate as a carbon source, because benzoate utilization, *β*-oxidation and non- *β*-oxidation phenotypes show the greatest up-regulation in both aromatic compound degrading conditions. It is known that *β*-oxidation is a key step in anaerobic degradation of aromatic compounds including the anaerobic benzoate degradation in *R. palustris* [29]. The involvement of non-β-oxidation route in the degradation *p*-coumarate was also recently reported [26]. Therefore, up-regulation of these phenotypes in combination with benzoate utilization phenotype indicate the presence of a *R. palustris* subpopulation utilizing benzoate as a carbon source under *p*-coumarate and benzoate degrading conditions.

The greater up-regualtion of *β*- and non-*β*-oxidations under *p*-coumarate degradation (Figure 1) if compared with benzoate degradation may be attributed to a subpopulation (or two different subpopulations) that utilize *p*-coumarate by *β*-oxidation and non-β-oxidation. This is consistent with a previous finding [26]. It is not clear, however, if the expression of *β*-oxidation and non-*β*-oxidation enzymes may coincide in one cell. Both subpopulations likely release 4-hydroxybenzoate or benzoate for subsequent utilization by another subpopulation. This release is consistent with a previous study of *R. palustris* growth on trans-cinnamate, which showed an initial utilization of cinnamate by *β*-oxidation [7]. The accumulation of 4-hydroxybenzoate was also observed in culture media during the *R. palustris* growth on *p*-coumarate [8].

The other two phenotypes that are common to the *R. palustris* growth on benzoate and *p*-coumarate are carbon dioxide and hydrogen gas utilization. We attributed the expression of these phenotypes to a photoautotrophic subpopulation of the bacterium. The utilization of CO_2_ by using hydrogen gas as a donor of electrons is a major characteristic of the photoautotrophic growth of *R. palustris*. Activation of genes representing uptake hydrogenase system in *R. palustris*, which are marker genes of H_2_ utilization, [12], is implausible for benzoate or *p*-coumarate utilizing subpopulations. Both hydrogen gas and benzoate/*p*-coumarate are used by *R. palustris* as electron donors. Therefore, assimilation of H_2_ would increase deficiency of electron acceptors for benzoate/*p*-coumarate oxidation in subpopulations utilizing these substrates. Although several reports indicated that nonsulfur purple photosynthetic bacteria can produce enzymes of the Calvin cycle to utilize the reduced carbon sources under photoheterotrophic growth conditions [19], [32]–[35], none of the reports demonstrated that utilization of carbon from both, CO_2_ and organic source, occurred simultaneously in the same bacterial cells and not in different subpopulations of the same bacterial organism.

Specific to the *R. palustris* growth on benzoate is the activation of nitrogen gas fixation and formate oxidation. We attributed these phenotypes to a nitrogen-fixing *R. palustris* subpopulation utilizing formate as a carbon source. The utilization of formate as a donor of electrons is also plausible for photoautotrophically growing *R. palustris*. The ability of *R. palustris* to metabolize formate growing photoautotrophically has been characterized by Qadri and Hoare [30].

**Figure 2:**
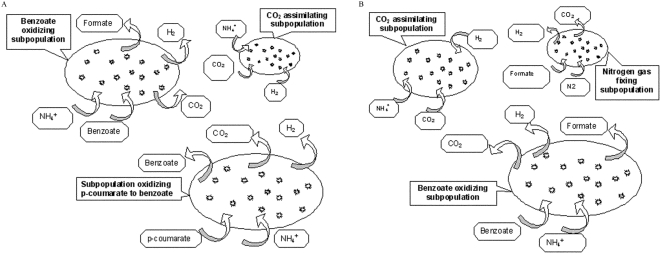
Structures of R. palustris consortia mediating anaerobic growth on p-coumarate (A) and on benzoate (B).

Based on identified subpopulations we hypothesize the following structures for a *p*-coumarate degrading consortium and a benzoate degrading consortium. The *R. palustris* consortium growing on *p*-coumarate as the only carbon source is comprised of at least three subpopulations utilizing *p*-coumarate (Syntroph 1), benzoate (Syntroph 2), CO_2_ and H_2_ (Syntroph 3) for their growth (Figure 2A). The *R. palustris* consortium growing on benzoate as the only carbon source also includes at least three subpopulations. Syntroph 1 oxidizes benzoate, Syntroph 2 assimilates CO_2_ by using H_2_ as an electron donor, and the N_2_-fixing Syntroph 3 utilizes formate as a carbon source (Figure 2B).

### Up-regulation of genes and proteins involved in chemotaxis, curli formation and transport reactions supports multi-subpopulation structure of the consortia

Utilization of different substrates by *R. palustris* subpopulations assumes a quick response of each subpopulation to the presence of the assimilating substrates in the environment and activity of different transport reactions.

Sensoring of substrates is important for all subpopulations in consortia. Therefore, genes/proteins involved in chemosensory pathways have great up-regulation in the benzoate/*p*-coumarate degrading conditions if compared with succinate. Although these pathways are the most studied in bacteria [36], *R. palustris* chemotaxis has not been studied in detail. Based on the *R. palustris* annotation [37], there are 52 genes in the genome potentially involved in chemotaxis. Thirty genes are clustered in 8 predicted transcription units. Expressions of genes and proteins involved in *R. palustris* chemotaxis (Table S3) demonstrates that most chemotaxis genes and proteins are at least 2-fold up-regulated when *R. palustris* is grown on benzoate/*p*-coumarate versus succinate. Average increase is 2.4-fold for genes and 2.8-fold for proteins.

Several up-regulated operons involved in chemotaxis are located immediately before clusters/operons of up-regulated genes annotated as predicted transporters (Table S3). One of the operons is comprised of 8 genes (*rpa0136- rpa0143*) with 2-4-fold up-regulation. The operon is followed by a predicted sulfate transporter, *rpa0144*. The transporter is 2-fold up-regulated during the growth on benzoate and on *p*-coumarate versus succinate. The up-regulation of the chemotaxis proteins and of the transporter suggests their involvement in the assimilation of sulfates, in both aromatic compound degrading growth conditions. The operon comprised of methyl-accepting chemotaxis receptors *rpa4638* and *rpa4639* is located immediately before the *cbb*_II_ operon involved in CO_2_ assimilation (*rpa4641- rpa4645*) (Table S3). Both operons are up-regulated during *R. palustris* growth on benzoate and *p*-coumarate; 4-fold (6-fold) gene (protein) expression increase is observed for *rpa4638* and *rpa4639*. High up-regulation of these chemotaxis transducer genes suggests that they may couple the activity of *cbb*_II_ operon with the availability of CO_2_ in the environment. Up-regulated predicted chemotaxis proteins MotA and MotB1 are followed by a putative potassium uptake protein Kup (RPA2011) involved in the export of K+. This transporter has 1.8-fold upregulation in the growth on *p*-coumarate and benzoate versus succinate. Another highly up-regulated operon of chemotaxis related genes (*rpa4302–rpa4307*) is located before a permease of the drug/metabolite transporter (DMT) superfamily (*rpa4299*). The transporter has 2.5-fold up-regulation in the growth on *p*-coumarate versus succinate and 2.5-fold down-regualtion in the growth on benzoate versus succinate. Thus, the operon and the transpoter may be involved in sensing and transporting of *p*-coumarate.

Curli formation is another important cellular process that helps bacterial adaptation to substrates availability in the environment. Curli are extracellular surface structures involved in cell-cell contacts and adherence to promote community behavior [38]. Based on *R. palustris* genome annotation and operon prediction, there are two clusters of genes in the genome that are potentially involved in the curli assembly (Table S3). These genes show almost 4-fold up-regulation during the *R. palustris* growth on *p*-coumarate and benzoate. This may indicate the formation of the curli in all subpopulations of the aromatic compounds degrading consortia. Increased adherence of *R. palustris* representing different subpopulations may be an important strategy to facilitate the movement of end-product from a subpopulation producing the product to a subpopulation consuming it.

### Nitrogen gas fixation under anaerobic benzoate degradation

Fingerprinting of phenotypes during growth on benzoate indicates a low, although statistically significant, up-regulation of the nitrogen gas fixation phenotype. Expression of the phenotype was calculated using 45 marker genes representing three nitrogenases with Mo-, Fe- and V cofactors studied in *R. palustris* by Oda et al. [27]. The referenced study shows that, although all three enzymes are functional, the level of their expression may be different. It depends on the levels of fixed nitrogen starvation and on the availability of the transition metals in the culture media. Thus, a difference in the average expression of marker genes representing different nitrogenases in the studied growth conditions may affect the expression level of the nitrogen gas fixation phenotype.

**Figure 3:**
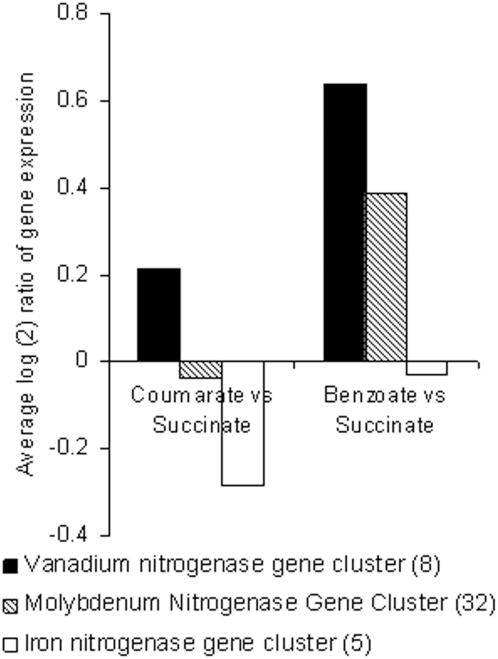
Average log2 ratio of the expression of nitrogenases with different cofactors in the growth on p-coumarate and benzoate versus succinate.

Indeed, we found that the expression of nitrogenases in the benzoate and *p*-coumarate versus succinate degrading conditions was essentially different (Figure 3). The nitrogenases with V and Mo cofactors have 1.6-fold (*p* = 0.00016) and 1.3-fold (*p* = 0.00056) increase in the average activity of the marker genes respectively. The Fe cofactor nitrogenase does not show any significant change in the expression. Considering the observed difference in the expression of the nitrogenases with different cofactors, we suggest that the screening of N_2_ fixation phenotype in the benzoate degrading condition using only marker genes for V cofactors nitrogenase would give a greater expression of the phenotype than is indicated in Figure 1. Therefore, the size of N_2_ fixing subpopulation in the benzoate degrading condition may be actually similar to the size of carbon dioxide utilizing subpopulation or greater.

### Experimental confirmation of nitrogen gas consumption by *R. palustris* grown on benzoate and ammonium

The consumption of nitrogen gas by *R. palustris* grown on benzoate with plenty of available ammonium in the culture was not reported before and may be debated. First, because fixed nitrogen is always the preferred N source for bacterial growth [39]. Second, because nitrogenase may be used by the bacterium to evolve hydrogen out of the cell and not for the N_2_ fixation, although this phenomenon was observed before only in mutant strains of the bacterium [22], [40]. In addition, the expression of the nitrogenases was found only at the level of genes, because detection of membrane proteins using liquid chromatography-mass spectrometry is more difficult [41]–[43].

**Figure 4:**
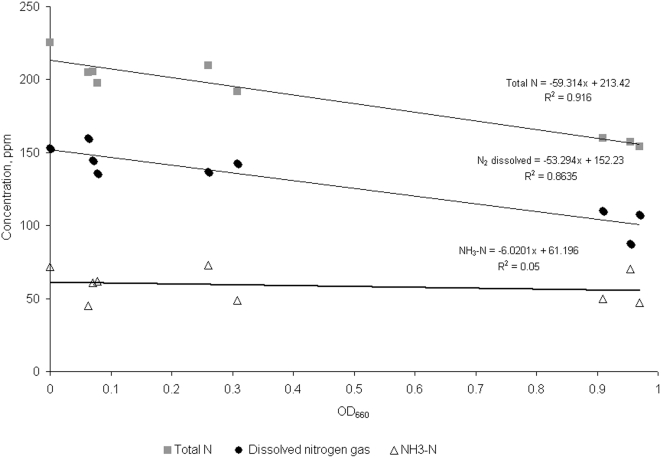
Changes in total nitrogen, ammonium and dissolved nitrogen gas during the benzoate degradation as functions of OD660.

To provide experimental confirmation of the N_2_ consumption, the growth of *R. palustris* on benzoate was reproduced under the same conditions using triplicate cultures, and changes in total nitrogen and ammonium were measured in the culture supernatant after centrifugation during the growth under different OD_660_. Given the only initial nitrogen sources in the culture were (NH_4_)_2_SO_4_ and dissolved nitrogen gas, concentration of the latter was calculated as difference between total N and ammonium. This difference, however, may also include any soluble form of nitrogen generated by consortium during the benzoate degradation. Figure 4 demonstrates a significant decrease of total N and dissolved nitrogen gas in course of degradation with correlation coefficients 0.95 and 0.93, respectively. Curiously, the ammonium doesn't show any decrease in concentration during the growth (R = 0.2). The results confirm the existence of nitrogen gas fixing subpopulation of *R. palustris* in the benzoate degrading conditions. They also suggest that this subpopulation produces ammonium as a product. This production refills the ammonium consumed by benzoate degrading subpopulation and by CO_2_ assimilating subpopulation. Indirect support for ammonium production and utilization gives a greater variability in the ammonium concentration across samples than variability of total and dissolved nitrogen (Figure 4).

## Discussion

Significant expression of several *R. palustris* phenotypes revealed by the phenotype fingerprinting approach in combination with up-regulation of genes and proteins involved in chemotaxis, curli formation and transport reactions indicates that this bacterium may anaerobically degrade a complex aromatic compound in light organizing a single-genotype consortium with each subpopulation implementing its own mode of metabolism. The emergence of phenotypically different, but genetically the same, subpopulations in *p*-coumarate or benzoate degrading *R. palustris* consortia under the studied conditions is likely been driving by the absence in the culture of efficient and readily available electron acceptors. The utilization of the reduced carbon sources, like benzoate, under anaerobic growth conditions generates a lot of reducing equivalents that must be oxidized in the cell by the consumed electron acceptors [19], [32], [33], [44], [45]. A shortage of the acceptors prevents complete degradation of the aromatic compounds in one bacterial cell by changing its redox potential and making it beneficial for the cell to release intermediate products in the environment for subsequent utilization by other subpopulations of the bacterial cells that are transcriptionally reprogrammed for distinct modes of the metabolism. The new modes may be triggered in some cells of the culture by changes in its oxidative conditions and by availability of CO_2_ and N_2_. Although these resources require a lot of energy for their utilization, they are very efficient electron acceptors. Thus, the emerged *R. palustris* subpopulations can balance the redox homeostasis in the culture environment as a whole and increase the total bacterial biomass by the new subpopulations outgrowth and by facilitating the anaerobic respiration of the benzoate utilizing subpopulation.

Although the involvement of subpopulations representing one bacterial organism in degradation of aromatic compounds was not reported before, the multi-organisms consortia utilizing hydrogen gas, carbon dioxide and fix nitrogen gas is a known phenomenon in natural environments enriched with recalcitrant carbon, even if the environment has high concentrations of ammonium. These populations, for example, were recently identified in microbial community specialized towards plant lignocellulose degradation in the hindgut of a wood-feeding higher termite [13]. CO_2_-reduction by H_2_ plays a central role in the fermentation of organic matter in anaerobic microbial ecosystems like swamps, anaerobic waste decomposition, and the intestinal tract [3], [15]. In marine sediments, for example, CO_2_-reduction occurs in addition to the reduction of sulfates that are typical for the environment [14]. Hydrogen utilization by the methanogens is also important in the reticulo-rumen where this process reduces the hydrogen partial pressure of the rumen and, thus, facilitate the metabolism of syntrophic hydrogen “donor” partner species [15]. The activation of nitrogen gas fixation by consortium of microbes is also reported in the environments enriched with complex organics. This phenomenon is very important for the maintenance of soil fertility. It is often observed in soil under aerobic and anaerobic conditions, especially when there is a high ratio of available C to available N [16]. It is also known that bacterial N_2_ fixation may take place in the anaerobic environments where concentrations of fixed nitrogen are high. For example, a relatively high rate of N_2_ fixation was found in anaerobic sediments with high concentrations of ammonium [39], [46]. Thus, the emerged subpopulations of *R. palustris* actually reproduce the natural bacterial consortiums utilizing recalcitrant carbon in diverse ecological and biological systems.

Our study does not provide direct evidence for the existence of the autotrophic and nitrogen gas fixing subpopulations. The proposed model of the *R. palustris* consortia is based on a computational analysis; additional experiments are necessary to validate it. Several factors can complicate an accurate detection of gene or protein expression in each subpopulation and the number of populations. First, up-regulation of a gene/protein in one subpopulation of the consortia may coincide with down-regulation of the gene/protein in another subpopulation. Second, the sizes of the populations are different, thus mRNAs/proteins of the dominating populations contribute more to the average expression. These factors may especially affect the identification of the activated processes in photoautotrophic and nitrogen-fixing subpopulations, which are likely less abundant. In addition, the number of subpopulations and their sizes may change in course of aromatic compound degradation according to changes in the available substrates. Hence, the time of the culture sampling may effect the identification of consortia structure. Initially the consortium is probably comprised of only one subpopulation and then it becomes more and more complex. This may explain the lack of N_2_-fixation subpopulation under the *p*-coumarate degrading condition, although this subpopulation is found under the benzoate degrading condition. In the analyzed studies, bacteria cells were sampled in the mid-log phase of the *R. palustris* growth on succinate, *p*-coumarate, or benzoate. Later sampling of the *p*-coumarate degrading culture would probably reveal the N*_2_*-fixing subpopulation as well. Later sampling of the benzoate degrading culture may also reveal some additional subpopulations. Studies of contaminated natural environments demonstrate an increasing microbial diversity of microbial communities during the course of degrading of the aromatic hydrocarbon contaminants [3]. Direct measurements of the gene/protein expression levels at single-cell resolution, such as single cell proteomics analysis [47] might be necessary for identification of the subpopulations.

The ability to form the single-genotype consortia may be a characteristic of the bacterial versatility, which is crucial for bacterial adaptation and gives the bacterium a significant evolutional advantage. Indeed, the consortium subpopulations do not compete, but complement each other. Specifically, they contribute to the production of consortia biomass and to the “insurance effects.” From the biomass production view-point, the exhaustion of main substrates in the consortia is supplemented by utilization of the end-products through the formation of new subpopulations, thus maintaining or increasing the total biomass. From the stand-point of the “insurance effects,” co-adhesion of functionally complementing cells, which is indicated in the study by curli formation, enhances bacterial survival, protects cells from injury, promotes communication between cells, and facilitates a coordinated behavior [48]. This set of complementary functions is a characteristic of a multicellular organism. Thus, the *R. palustris* consortia with subpopulations that are transcriptionally reprogrammed for different modes of metabolism is akin to the multicellular organism whose cells have the same genomes but differentiated in different ways to form different tissues. Similar to a tissue in the multicellular organism, each subpopulation in the consortium implements its own mode of growth contributing to development of whole bacterial organism.

In conclusion, the study demonstrates a utility of the proposed bioinformatics approach based on fingerprinting of bacterial phenotypes for analysis of large-scale transcriptomics and proteomics data. Using this approach we have revealed potential subpopulations of *R. palustris* involved in anaerobic degradation of benzoate and *p*-coumarate. The subpopulations may be produced by transcriptional reprogramming of the *R. palustris* genome for activation of the phenotypes that reduce efforts for maintenance of the redox homeostasis during the aromatic compound degradation. A single-genotype consortium is a novel phenomenon, and may be found not only under condition of aromatic compounds degradation. The study opens up avenues of research for characterization of the phenomenon and underlying mechanisms.

## Materials and Methods

### Experimental procedure

The experimental procedure described by Pan et al. [26] in more detail was used in the study of benzoate and *p*-coumarate degradation and the same procedure was also repeated for experimental confirmation of nitrogen gas consumption in this study. Briefly, wild type *R. palustris* CGA0010 was grown anaerobically on different defined mineral growth media in sealed tubes with a nitrogen gas headspace under pressure. The growth was at 30°C with ample incandescent light illumination. (NH_4_)_2_SO_4_ was the only source of fixed nitrogen available for bacterial assimilation, and was provided as (^14^NH_4_)_2_SO_4_ for the unlabeled culture and as (^15^NH_4_)_2_SO_4_ for the ^15^N-labeled culture (>98 atom percentage excess, Sigma-Aldrich, St. Louis, MO). 3 mM *p*-coumarate was supplied as the sole organic carbon source for the unlabeled coumarate culture. 3 mM benzoate or 10 mM succinate were supplied as the sole organic carbon sources for the ^15^N-labeled benzoate and succinate cultures, respectively. Duplicate cultures were prepared for each of the three growth conditions. Cell growth was monitored spectrophotometrically at 660 nm and cells were harvested in mid-log phase at OD_660 nm_ of 0.6 by centrifugation and washed twice with ice-cold wash buffer (50 mM Tris-HCl buffer at pH 7.5 with 10 mM EDTA). The harvested cell pellet from each culture was divided for quantitative shotgun measurements and microarray analysis.

Growth conditions for monitoring changes in total nitrogen and ammonium during the growth of *R. palustris* on benzoate was similar to described above, but triplicate cultures were prepared for the experiment. Forty ml samples were collected during the cultures growth at different OD_660_. Culture supernatant after centrifugation of each sample was used for chemical analyses.

### Chemical analyses

Total nitrogen was determined by combustion method using chemiluminescence gas analyzer TOC-V CPH/TPN Shimadzu [49]. The method provided 99.1% recovery of total nitrogen. Ammonium was measured colorimetrically using Nessler's reagent [50].

### Statistical analysis of the preprocessed data

In the present study preprocessed microarray and proteomics measurements of mRNA and protein expression were employed to reveal phenotypes activated in the *R. palustris* growth on *p*-coumarate (a hydroxyl derivative of cinnamic acid) and benzoate versus the *R. palustris* growth on succinate. The conditions are referred as the *p*-coumarate degrading condition (Cou vs Suc) and the benzoate degrading condition (Ben vs Suc). Results of the previous *R. palustris* studies were employed to collect biological information on marker genes representing different *R. palustris* phenotypes (Table S1). Average expression of the marker genes and proteins in a cluster/operon calculated in the dataset was used as a characteristic of the phenotype activity in the condition represented by the dataset. Statistical significance of the average mRNA/protein expression in the cluster/operon was estimated by *p*-value in the heteroscedastic *t*-test (two-samples with unequal variance). This test compares means between two samples using their standard deviations. The samples were considered to be independent. The first sample in this test is the phenotype-representing cluster/operon. It was characterized by the mean and by the standard deviation calculated for the cluster/operon from gene/protein log_2_-ratio in the dataset. The second sample represented a randomly sampled cluster/operon with the same number of genes as the phenotype-representing cluster/operon. To find the mean and the standard deviation for the second sample, we produced 1000 random gene clusters comprised of the same number of genes as the phenotype-representing cluster. Then we calculated the average for these clusters and the standard deviation based on the log_2_-ratio of gene/protein expression in the dataset. Significance of the difference between means in two samples was estimated by the *t*-test *p*-value. Thus, the *p*-values referred in the text and tables of the paper give probabilities that we get our observed difference between the means of the phenotype-representing cluster/operon and the randomly sampled cluster/operon by chance alone. The algorithm was implemented as a Visual Basic application in Excel. Predicted operons for the analysis were downloaded from the BioCyc *R. palustris* database [37]. The collected and downloaded biological information was integrated in the analysis using MS Access and MySQL.

## Supporting Information

Figure S1 Distribution of the levels of gene expressions (log(2) ratios) in the benzoate degrading condition versus succinate degrading condition
(0.07 MB PDF)

Table S1 Marker genes representing known R. palustris phenotypes
(0.08 MB DOC)

Table S2 Calculated p-values for expression of known R. palustris phenotypes and individual expressions of marker genes and proteins
(0.03 MB XLS)

Table S3 Expression of genes and proteins involved in chemotaxis, transport reactions and curli formation under aromatic compounds degrading conditions.
(0.25 MB DOC)

## References

[1] Zaldivar J, Nielsen J, Olsson L (2001). Fuel ethanol production from lignocellulose: a challenge for metabolic engineering and process integration.. Appl Microbiol Biotechnol.

[2] Samanta SK, Singh OV, Jain RK (2002). Polycyclic aromatic hydrocarbons: environmental pollution and bioremediation.. Trends Biotechnol.

[3] Zwolinski, Harris RF, Hickey WJ (2000). Microbial consortia involved in the anaerobic degradation of hydrocarbons.. Biodegradation.

[4] Becker JG, Berardesco G, Rittmann BE, Stahl DA (2005). The role of syntrophic associations in sustaining anaerobic mineralization of chlorinated organic compounds.. Environ Health Perspect.

[5] Ficker M, Krastel K, Orlicky S, Edwards E (1999). Molecular characterization of a toluene-degrading methanogenic consortium.. Appl Environ Microbiol.

[6] Gibson J, C SH (2002). Metabolic diversity in aromatic compound utilization by anaerobic microbes.. Annu Rev Microbiol.

[7] Elder DJ, Morgan P, Kelly DJ (1992). Anaerobic degradation of trans-cinnamate and omega-phenylalkane carboxylic acids by the photosynthetic bacterium Rhodopseudomonas palustris: evidence for a beta-oxidation mechanism.. Arch Microbiol.

[8] Harrison FH (2005). Peripheral pathways of anaerobic benzoate degradation in Rhodopseudomonas palustris.

[9] Heider J, Fuchs G (1997). Anaerobic metabolism of aromatic compounds.. Eur J Biochem.

[10] Harwood CSBG, Herrmann H, Fuchs G (1999). Anaerobic metabolism of aromatic compounds via the benzoyl-CoA pathway.. FEMS Microbiol Rev.

[11] VerBerkmoes NC, Shah MB, Lankford PK, Pelletier DA, Strader MB (2006). Determination and comparison of the baseline proteomes of the versatile microbe Rhodopseudomonas palustris under its major metabolic states.. J Proteome Res.

[12] Rey FE, Oda Y, Harwood CS (2006). Regulation of uptake hydrogenase and effects of hydrogen utilization on gene expression in Rhodopseudomonas palustris.. J Bacteriol.

[13] Warnecke F, Luginbuhl P, Ivanova N, Ghassemian M, Richardson TH (2007). Metagenomic and functional analysis of hindgut microbiota of a wood-feeding higher termite.. Nature.

[14] Oremland RS, Taylor BF (1978). Sulfate reduction and methanogenesis in marine sediments.. Geochimica et Cosmochimica Acta.

[15] Wolin MJ, Miller TL (1982). Interspecies hydrogen transfer: 15 years later.. ASM American Society for Microbiology News.

[16] Weber A, Sundman V (1986). Nitrogen fixation in coniferous bark litter.. Plant and Soil.

[17] Fisher SH, Sonenshein AL (1991). Control of carbon and nitrogen metabolism in Bacillus subtilis.. Annu Rev Microbiol.

[18] Bruckner R, Titgemeyer F (2002). Carbon catabolite repression in bacteria: choice of the carbon source and autoregulatory limitation of sugar utilization.. FEMS Microbiol Lett.

[19] Wang X, Falcone DL, Tabita FR (1993). Reductive pentose phosphate-independent CO2 fixation in Rhodobacter sphaeroides and evidence that ribulose bisphosphate carboxylase/oxygenase activity serves to maintain the redox balance of the cell.. J Bacteriol.

[20] D Karl AM, Bergman B, Capone D, Carpenter E, Letelier R, Lipschultz F, Paerl H, Sigman D, Stal L (2004). Dinitrogen fixation in the world's oceans.

[21] Larimer FW, Chain P, Hauser L, Lamerdin J, Malfatti S (2004). Complete genome sequence of the metabolically versatile photosynthetic bacterium Rhodopseudomonas palustris.. Nat Biotechnol.

[22] Joshi HM, Tabita FR (1996). A global two component signal transduction system that integrates the control of photosynthesis, carbon dioxide assimilation, and nitrogen fixation.. Proc Natl Acad Sci U S A.

[23] Qian Y, Tabita FR (1998). Expression of glnB and a glnB-like gene (glnK) in a ribulose bisphosphate carboxylase/oxygenase-deficient mutant of Rhodobacter sphaeroides.. J Bacteriol.

[24] Barbosa MJ, Rocha JM, Tramper J, Wijffels RH (2001). Acetate as a carbon source for hydrogen production by photosynthetic bacteria.. J Biotechnol.

[25] Sawayama S, Tsukahara K, Yagishita T, Hanada S (2001). Characterization of lighted upflow anaerobic sludge blanket (LUASB) method under sulfate-rich conditions.. J Biosci Bioeng.

[26] Pan C, Oda Y, Lankford PK, Zhang B, Samatova NF (2007). Characterization of anaerobic catabolism of p-coumarate in rhodopseudomonas palustris by integrating transcriptomics and quantitative proteomics.. Molecular&Cellular Proteomics.

[27] Oda Y, Samanta SK, Rey FE, Wu L, Liu X (2005). Functional genomic analysis of three nitrogenase isozymes in the photosynthetic bacterium Rhodopseudomonas palustris.. J Bacteriol.

[28] Romagnoli S, Tabita FR (2006). A novel three-protein two-component system provides a regulatory twist on an established circuit to modulate expression of the cbbI region of Rhodopseudomonas palustris CGA010.. J Bacteriol.

[29] Harrison FH, Harwood CS (2005). The pimFABCDE operon from Rhodopseudomonas palustris mediates dicarboxylic acid degradation and participates in anaerobic benzoate degradation.. Microbiology.

[30] Qadri SM, Hoare DS (1968). Formic hydrogenlyase and the photoassimilation of formate by a strain of Rhodopseudomonas palustris.. J Bacteriol.

[31] Barassi CA, Kranz RG, Gennis RB (1985). Succinate dehydrogenase in Rhodopseudomonas sphaeroides: subunit composition and immunocross-reactivity with other related bacteria.. J Bacteriol.

[32] Falcone DL, Tabita FR (1991). Expression of endogenous and foreign ribulose 1,5-bisphosphate carboxylase-oxygenase (RubisCO) genes in a RubisCO deletion mutant of Rhodobacter sphaeroides.. J Bacteriol.

[33] Hallenbeck PL, Lerchen R, Hessler P, Kaplan S (1990). Roles of CfxA, CfxB, and external electron acceptors in regulation of ribulose 1,5-bisphosphate carboxylase/oxygenase expression in Rhodobacter sphaeroides.. J Bacteriol.

[34] McEwan AG (1994). Photosynthetic electron transport and anaerobic metabolism in purple non-sulfur phototrophic bacteria.. Antonie Van Leeuwenhoek.

[35] Tabita FR (1988). Molecular and cellular regulation of autotrophic carbon dioxide fixation in microorganisms.. Microbiol Rev.

[36] Wadhams GH, Armitage JP (2004). Making sense of it all: bacterial chemotaxis.. Nat Rev Mol Cell Biol.

[37] Karp PD, Ouzounis CA, Moore-Kochlacs C, Goldovsky L, Kaipa P (2005). Expansion of the BioCyc collection of pathway/genome databases to 160 genomes.. Nucleic Acids Res.

[38] Barnhart MM, Chapman MR (2006). Curli biogenesis and function.. Annu Rev Microbiol.

[39] Howarth RW, Marino R, Cole JJ (1988). Nitrogen Fixation in Freshwater, Estuarine, and Marine Ecosystems. 2. Biogeochemical Controls.. Limnology and Oceanography.

[40] Rey FE, Heiniger EK, Harwood CS (2007). Redirection of metabolism for biological hydrogen production.. Appl Environ Microbiol.

[41] Chen L, Deng L, Liu L, Peng Z (2007). Immunomagnetic separation and MS/SPR end-detection combined procedure for rapid detection of Staphylococcus aureus and protein A.. Biosens Bioelectron.

[42] Thompson MR, Chourey K, Froelich JM, Erickson BK, Verberkmoes NC (2008). Experimental Approach for Deep Proteome Measurements from Small-Scale Microbial Biomass Samples.. Anal Chem.

[43] Varga AR, Staehelin LA (1983). Spatial differentiation in photosynthetic and non-photosynthetic membranes of Rhodopseudomonas palustris.. J Bacteriol.

[44] Kasai Y, Takahata Y, Hoaki T, Watanabe K (2005). Physiological and molecular characterization of a microbial community established in unsaturated, petroleum-contaminated soil.. Environ Microbiol.

[45] Boll M (2005). Dearomatizing benzene ring reductases.. J Mol Microbiol Biotechnol.

[46] Howarth RW, Marino R, Lane J, Cole JJ (1988). Nitrogen Fixation in Freshwater, Estuarine, and Marine Ecosystems. 1. Rates and Importance.. Limnology and Oceanography.

[47] Newman JR, Ghaemmaghami S, Ihmels J, Breslow DK, Noble M (2006). Single-cell proteomic analysis of S. cerevisiae reveals the architecture of biological noise.. Nature.

[48] Castonguay MH, van der Schaaf S, Koester W, Krooneman J, van der Meer W (2006). Biofilm formation by Escherichia coli is stimulated by synergistic interactions and co-adhesion mechanisms with adherence-proficient bacteria.. Res Microbiol.

[49] Helrick K Official methods of analysis. sl; AOAC, 1990, 1422 p 2 v Ilus Contents 1

[50] Clesceri LS, Greenberg AE, Eaton AD (1998). Standard methods for the examination of water and wastewater.

