# Supplementary figures and images for: Phenotype Fingerprinting Suggests the Involvement of Single-Genotype Consortia in Degradation of Aromatic Compounds by Rhodopseudomonas palustris
Source: PLoS One. 2009 Feb 26;4(2):e4615. doi: 10.1371/journal.pone.0004615 (PMC2643473; doi:10.1371/journal.pone.0004615)

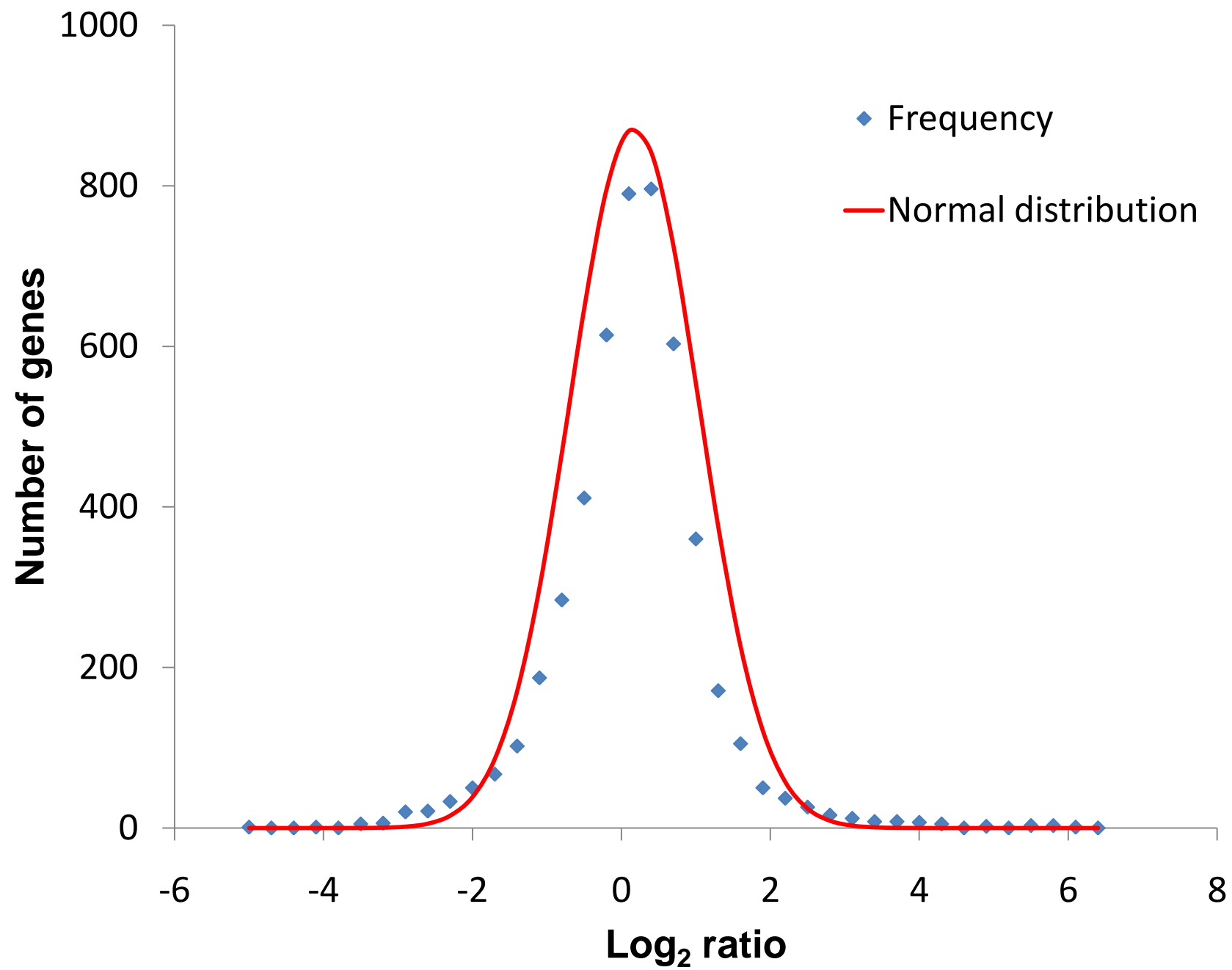

Supplement: Figure S1 — Distribution of the levels of gene expressions (log(2) ratios) in the benzoate degrading condition versus succinate degrading condition (0.07 MB PDF) [file pone.0004615.s001.pdf]
