# Supplementary material for: Phenotype Fingerprinting Suggests the Involvement of Single-Genotype Consortia in Degradation of Aromatic Compounds by Rhodopseudomonas palustris
Source: PLoS One. 2009 Feb 26;4(2):e4615. doi: 10.1371/journal.pone.0004615 (PMC2643473; doi:10.1371/journal.pone.0004615)
Supplement: Table S1 — Marker genes representing known R. palustris phenotypes (0.08 MB DOC) [file pone.0004615.s002.doc]

Table S1. Marker genes representing known *R. palustris* phenotypes.

| Phenotype | Marker genes | Comments | Ref. |
| --- | --- | --- | --- |
| Benzoate utilization | badD, badI, badK, badB, badG, badF, badH, badA, badE | Enzymes involved in CoA ligation, ring reduction, and ring cleavage (beta-oxidation step is not included, see pim-cluster below) | [1] |
| Beta oxidation | pimE, pimD, pimC, pimB, pimA, pimF | Enzymes representing beta oxidation-route | [2] |
| Non-beta oxidation | rpa1786, fcs2, rpa1206, hbaA | Enzymes representing non-beta-oxidation route | [1] |
| Carbon dioxide utilization | cbbR, cbbRR1, cbbRR2, cbbSR, cbbL, cbbS, cbbX, rpa1562, cbbM, cbbA, cbbT1, cbbP, cbbF | cbbI and cbbII gene clusters representing enzymes of the Calvin cycle | [3] |
| Nitrogen fixation | fixX, fixC, fixB, fixA, nifW, nifV, nifS2, nifU, rpa4610, nifQ, fdxB, rpa4613, rpa4614, nifX, nifN, nifE, nifK, nifD, nifH4, rpa4621, rpa4622, nifT, rpa4624, nifZ, rpa4626, rpa4627, hesB, fern, nifB, fer1, nifA, fixR2, anfA, anfD, anfG, anfH, anfK, vnfA, vnfD, vnfE, vnfG, vnfH, vnfK, vnfN, vnfX | Three gene clusters representing three nitrogenases with Mo-, Fe- and V cofactors | [4] |
| Hydrogen gas utilization | hupU, hupS, hupL, hupC, hupD, hupE, hupF, hupG, hupH, hupJ, hupK | Structural and accessory proteins of the uptake hydrogenase | [5] |
| Formate utilization | fdsG, fdsB, fdsA, fdsC, fdsD, rpa3201 | Subunits of NAD-dependent formate dehydrogenase (fdsABCDG operon) and a predicted transporter | [5,6] |
| Aerobic growth in the dark | rpa0501, rpa0580,rpa0738, gst2, coxB, rpa1741, rpa1763, mutB, rpa1846, rpa1851, rpa1876, rpa2120, rpa2125, rpa2126, rpa2269, rpa2471, rpa2473, rpa2875, rpa3188, rpa3440, rpa3466, rpa3470, rpa3471, fiu, fumC2, rpa3693, lytB1, rpa3915, rpa3930, fbpA, rpa4179 | Cluster of activated genes | [7] |
| Succinate utilization | rpa0216, rpa0217, rpa0218, rpa0219 | Succinate dehydrogenase (sdhABCD operon) | [8] |

1. Pan C, Oda Y, Lankford PK, Zhang B, Samatova NF, et al. (2007) Characterization of anaerobic catabolism of p-coumarate in rhodopseudomonas palustris by integrating transcriptomics and quantitative proteomics. Molecular & Cellular Proteomics 7: 938-948.

2. Harrison FH, Harwood CS (2005) The pimFABCDE operon from Rhodopseudomonas palustris mediates dicarboxylic acid degradation and participates in anaerobic benzoate degradation. Microbiology 151: 727-736.

3. Romagnoli S, Tabita FR (2006) A novel three-protein two-component system provides a regulatory twist on an established circuit to modulate expression of the cbbI region of Rhodopseudomonas palustris CGA010. J Bacteriol 188: 2780-2791.

4. Oda Y, Samanta SK, Rey FE, Wu L, Liu X, et al. (2005) Functional genomic analysis of three nitrogenase isozymes in the photosynthetic bacterium Rhodopseudomonas palustris. J Bacteriol 187: 7784-7794.

5. Rey FE, Oda Y, Harwood CS (2006) Regulation of uptake hydrogenase and effects of hydrogen utilization on gene expression in Rhodopseudomonas palustris. J Bacteriol 188: 6143-6152.

6. Qadri SM, Hoare DS (1968) Formic hydrogenlyase and the photoassimilation of formate by a strain of Rhodopseudomonas palustris. J Bacteriol 95: 2344-2357.

7. VerBerkmoes NC, Shah MB, Lankford PK, Pelletier DA, Strader MB, et al. (2006) Determination and comparison of the baseline proteomes of the versatile microbe Rhodopseudomonas palustris under its major metabolic states. J Proteome Res 5: 287-298.

8. Barassi CA, Kranz RG, Gennis RB (1985) Succinate dehydrogenase in Rhodopseudomonas sphaeroides: subunit composition and immunocross-reactivity with other related bacteria. J Bacteriol 163: 778-782.
