# Supplementary material for: Phenotype Fingerprinting Suggests the Involvement of Single-Genotype Consortia in Degradation of Aromatic Compounds by Rhodopseudomonas palustris
Source: PLoS One. 2009 Feb 26;4(2):e4615. doi: 10.1371/journal.pone.0004615 (PMC2643473; doi:10.1371/journal.pone.0004615)
Supplement: Table S3 — Expression of genes and proteins involved in chemotaxis, transport reactions and curli formation under aromatic compounds degrading conditions. (0.25 MB DOC) [file pone.0004615.s004.doc]

Table S3. Expression of genes and proteins involved in chemotaxis, CO_2_ assimilation (*cbb_I_ and cbb_Ii_* clusters), curli formation, aerobic respiration (*fix* gene cluster and *ccoPQON* operon), ammonium and nitrogen regulation (pII genes cluster) under conditions of p-coumarate and benzoate degradation if compared with *R. palustris* growth on succinate (log_2_-ratio). Predicted operons are shaded in different colors.

| Gene ID | Protein Name | | | Gene Name | | mRNAs | | | | Proteins | | | |
| --- | --- | --- | --- | --- | --- | --- | --- | --- | --- | --- | --- | --- | --- |
|  |  |  |  |  |  | Cou  vs  Suc | | | Ben vs Suc | Cou  vs  Suc | | | Ben vs Suc |
| **Gene clusters and operons involved in chemotaxis** | | | | | | | | | | | | | |
| RPA0136 | methyl-accepting chemotaxis receptor/sensory transducer | | | *rpa0136* | | 0.98 | | | 0.85 |  | | |  |
| RPA0137 | chemotaxis methylesterase, CheB1 | | | *cheB1* | | 0.78 | | | 1.35 | 0.84 | | | 1.32 |
| RPA0138 | chemotaxis methyltransferase, CheR1 | | | *cheR1* | | 1.28 | | | 1.35 |  | | |  |
| RPA0139 | methyl-accepting chemotaxis receptor/sensory transducer | | | *rpa0139* | | 1.18 | | | 2.15 | 1.34 | | | 2.42 |
| RPA0140 | chemotaxis signal transduction/oligomerization protein CheW1-2 | | | *cheW1-3* | | 1.48 | | | 1.85 |  | | |  |
| RPA0141 | chemotaxis signal transduction/oligomerization protein CheW1-1 | | | *cheW1-1* | | 1.38 | | | 1.75 |  | | |  |
| RPA0142 | multidomain chemotaxis histidine kinase CheA1 | | | *cheA1* | | 1.18 | | | 1.55 | 1.44 | | | 2.12 |
| RPA0143 | response regulator receiver, CheY1 | | | *cheY1* | | -0.38 | | | 1.35 | -0.89 | | | 1.92 |
| RPA0234 | methyl-accepting chemotaxis receptor/sensory transducer | | | *rpa0234* | | -0.22 | | | 0.35 |  | | |  |
| RPA0431 | methyl-accepting chemotaxis receptor/sensory transducer with PAS domain | | | *rpa0431* | | -1.32 | | | -0.75 | -1.76 | | | -1.68 |
| RPA0856 | methyl-accepting chemotaxis receptor/sensory transducer | | | *rpa0856* | | -0.72 | | | -0.75 |  | | |  |
| RPA1096 | methyl-accepting chemotaxis sensory transducer | | | *rpa1096* | | 3.68 | | | 2.85 |  | | |  |
| RPA1629 | chemotaxis response regulator, CheY2 | | | *cheY2* | | 0.78 | | | 1.35 | 0.44 | | | 1.02 |
| RPA1630 | chemotaxis methylesterase, CheB2 | | | *cheB2* | | 0.18 | | | 0.75 | 0.04 | | | 0.32 |
| RPA1631 | chemotaxis methyltransferase, CheR2 | | | *cheR2* | | 0.88 | | | 0.85 |  | | |  |
| RPA1640 | methyl-accepting chemotaxis receptor/sensory transducer | | | *rpa1640* | | -0.62 | | | -0.45 |  | | |  |
| RPA1675 | methyl-accepting chemotaxis receptor/sensory transducer with PAS domain | | | *rpa1675* | | 0.18 | | | 0.45 |  | | |  |
| RPA1678 | chemotaxis methyltransferase, CheR3 | | | *cheR3* | | 1.98 | | | 2.35 | 1.44 | | | 2.12 |
| RPA1822 | methyl-accepting chemotaxis receptor/sensory transducer | | | *rpa1822* | | 1.68 | | | 1.85 |  | | |  |
| RPA1850 | methyl-accepting chemotaxis receptor/sensory transducer | | | *rpa1850* | | -0.22 | | | 0.05 |  | | |  |
| RPA1884 | methyl-accepting chemotaxis receptor/sensory transducer | | | *rpa1884* | | 2.48 | | | 2.85 |  | | |  |
| RPA1931 | methyl-accepting chemotaxis receptor/sensory transducer | | | *rpa1931* | | 0.18 | | | -0.05 | -0.16 | | |  |
| RPA2009 | possible chemotaxis protein MotA | | | *rpa2009* | | 0.48 | | | 0.85 |  | | |  |
| RPA2010 | putative chemotaxis protein MotB1 | | | *motB1* | | 0.58 | | | 0.75 |  | | |  |
| RPA2479 | methyl-accepting chemotaxis receptor/sensory transducer | | | *rpa2479* | | 0.08 | | | 0.55 |  | | |  |
| RPA2897 | possible chemotaxis protein MotB2 | | | *motB2* | | 0.28 | | | 0.65 |  | | |  |
| RPA3185 | methyl-accepting chemotaxis receptor/sensory transducer | | | *rpa3185* | | 2.68 | | | 3.35 | 2.74 | | | 3.52 |
| RPA3316 | possible chemotaxis CheB/CheR fusion protein | | | *rpa3316* | | 0.18 | | | 0.05 |  | | |  |
| RPA3546 | methyl-accepting chemotaxis receptor/sensory transducer | | | *rpa3546* | | 0.88 | | | 0.95 | 0.64 | | | 1.02 |
| RPA3598 | methyl-accepting chemotaxis receptor/sensory transducer | | | *rpa3598* | | -0.12 | | | -0.55 | 0.14 | | | 0.52 |
| RPA3750 | methyl-accepting chemotaxis receptor/sensory transducer | | | *rpa3750* | | 2.78 | | | 2.85 |  | | |  |
| RPA4202 | methyl-accepting chemotaxis sensory transducer | | | *rpa4202* | | 0.98 | | | 0.05 |  | | |  |
| RPA4203 | putative sensor (PAS) domain for methyl-accepting chemotaxis sensory transducer | | | *rpa4203* | | 0.18 | | | 0.35 |  | | |  |
| RPA4302 | methyl-accepting chemotaxis receptor/sensory transducer | | | *rpa4302* | | 2.08 | | | 2.65 | 1.34 | | | 2.02 |
| RPA4303 | orf | | | *rpa4303* | | -0.08 | | | 0.35 |  | | |  |
| RPA4304 | orf | | | *rpa4304* | | -1.18 | | | 2.05 |  | | |  |
| RPA4305 | orf | | | *rpa4305* | | 0.12 | | | 1.05 |  | | |  |
| RPA4306 | methyl-accepting chemotaxis receptor/sensory transducer | | | *rpa4306* | | 0.98 | | | 1.05 | 0.44 | | | 0.82 |
| RPA4307 | methyl-accepting chemotaxis receptor/sensory transducer | | | *rpa4307* | | 0.18 | | | 0.15 |  | | |  |
| RPA4310 | orf | | | *rpa4310* | | -0.18 | | | 1.85 |  | | |  |
| RPA4311 | methyl-accepting chemotaxis sensory transducer | | | *rpa4311* | | 2.18 | | | 2.55 |  | | |  |
| RPA4312 | putative sensor (PAS) domain for methyl-accepting chemotaxis sensory transducer | | | *rpa4312* | | 2.68 | | | 3.45 |  | | |  |
| RPA4449 | pmethyl-accepting chemotaxis receptor/sensory transducer | | | *rpa4449* | | -0.62 | | | -0.35 | 0.24 | | | -0.08 |
| RPA4481 | methyl-accepting chemotaxis sensory transducer | | | *rpa4481* | | 0.88 | | | 0.75 |  | | |  |
| RPA4482 | putative sensor (PAS) domain for methyl-accepting chemotaxis sensory transducer | | | *rpa4482* | | 0.48 | | | 1.35 |  | | |  |
| RPA4483 | methyl-accepting chemotaxis sensory transducer | | | *rpa4483* | | -0.12 | | | 0.55 |  | | |  |
| RPA4484 | putative sensor (PAS) domain for methyl-accepting chemotaxis sensory transducer | | | *rpa4484* | | 0.98 | | | 1.15 |  | | |  |
| RPA4638 | methyl-accepting chemotaxis receptor/sensory transducer | | | *rpa4638* | | 0.38 | | | 0.65 | 0.54 | | | 1.02 |
| RPA4639 | methyl-accepting chemotaxis receptor/sensory transducer | | | *rpa4639* | | 2.98 | | | 3.75 | 3.64 | | | 4.42 |
| RPA4663 | methyl-accepting chemotaxis receptor/sensory transducer | | | *rpa4663* | | 0.88 | | | 1.35 |  | | |  |
| RPA4684 | methyl-accepting chemotaxis receptor/sensory transducer | | | *rpa4684* | | 3.18 | | | 4.05 |  | | |  |
| RPA4691 | methyl-accepting chemotaxis receptor/sensory transducer | | | *rpa4691* | | 2.68 | | | 2.95 |  | | |  |
| Average |  | | |  | | 0.85 | | | 1.25 | 0.73 | | | 1.43 |
| P-value |  | | |  | | 1.0E-05 | | | 1.7E-09 | 3.0E-02 | | | 1.4E-03 |
|  | | | | | | | | | | | | | |
| **CO_2_ assimilation gene clusters** | | | | | | | | | | | | | |
| *cbb_I_* cluster | | | | | | | | | | | | | |
| RPA1555 | *cbb* operon transcriptional regulator CbbR, LysR family | | | *cbbR* | | -0.42 | | 0.15 | | |  | |  |
| RPA1556 | phosphotransfer protein with response regulator receiver and Hpt domains | | | *cbbRR1* | | 3.58 | | 5.35 | | |  | |  |
| RPA1557 | response regulator receiver with duplicated domains | | | *cbbRR2* | | 1.78 | | 2.65 | | |  | |  |
| RPA1558 | sensor histidine kinase with multiple PAS/PAC and a response regulator receiver domain | | | *cbbSR* | | 1.78 | | 2.45 | | | -0.36 | | -0.28 |
| RPA1559 | ribulose-bisphosphate carboxylase large chain | | | *cbbL* | | 3.88 | | 2.45 | | | 2.24 | | 3.02 |
| RPA1560 | ribulose-bisphosphate carboxylase small chain | | | *cbbS* | | 4.58 | | 3.25 | | | 2.04 | | 2.72 |
| RPA1561 | CbbX protein homolog | | | *cbbX* | | 4.48 | | 3.05 | | | 1.54 | | 2.32 |
| RPA1562 | transcriptional regulator, LysR family | | | *rpa1562* | | 3.68 | | 1.35 | | |  | |  |
| Average |  | | |  | | 2.92 | | 2.59 | | |  | |  |
| P-value |  | | |  | | 1.9E-03 | | 1.8E-03 | | | 1.37 | | 1.95 |
| RPA4640 | orf | | | *rpa4640* | | 1.58 | | 1.85 | | | 1.0E-01 | | 7.9E-02 |
| *cbb_II_* cluster | | | | | | | | | | | | | |
| RPA4641 | ribulose-bisphosphate carboxylase form II | *cbbM* | 1.28 | | 1.05 | | 1.04 | | | | | 1.22 | |
| RPA4642 | fructose-bisphosphate aldolase | *cbbA* | 1.48 | | 1.15 | | 1.14 | | | | | 1.32 | |
| RPA4643 | transketolase | *cbbT1* | 1.58 | | 0.95 | | 1.24 | | | | | 1.42 | |
| RPA4644 | phosphoribulokinase | *cbbP* | 1.58 | | -0.15 | | 1.24 | | | | | 1.32 | |
| RPA4645 | fructose-1,6-bisphosphatase | *cbbF* | 0.78 | | -0.75 | | 1.74 | | | | | 1.82 | |
| Average |  |  | 1.34 | | 0.45 | | 1.28 | | | | | 1.42 | |
| P-value |  |  | 9.1E-04 | | 3.1E-01 | | 5.2E-04 | | | | | 1.9E-04 | |
|  | | | | | | | | | | | | | |
| **Curli formation clusters** | | | | | | | | | | | | | |
| RPA1083 | orf | *rpa1083* | 1.68 | | 2.05 | |  | | | | |  | |
| RPA1084 | possible minor curlin subunit precursor (fimbrin sef17 minor subunit). | *csgB* | 2.48 | | 1.95 | |  | | | | |  | |
| RPA1085 | orf | *rpa1085* | 1.58 | | 1.85 | |  | | | | |  | |
| RPA1086 | possible curli production assembly/transport component csgg precursor | *csgG* | 2.68 | | 2.25 | |  | | | | |  | |
| RPA1087 | possible transglycosylase SLT domain | *rpa1087* | 1.88 | | 1.75 | |  | | | | |  | |
|  | Average |  | 2.06 | | 1.97 | |  | | | | |  | |
|  | P-value |  | 7.4E-04 | | 2.0E-05 | |  | | | | |  | |
| RPA3330 | orf | *rpa3330* | 1.78 | | -0.95 | |  | | | | |  | |
| RPA3331 | orf | *rpa3331* | 0.68 | | 1.25 | |  | | | | |  | |
| RPA3332 | orf | *rpa3332* | 1.78 | | 1.75 | |  | | | | |  | |
| RPA3333 | putative curli production assembly/transport component csgg precursor | *csgG* | 2.58 | | 2.25 | |  | | | | |  | |
|  | Average (rpa3330 is not included) |  | 1.68 | | 1.75 | |  | | | | |  | |
|  | P-value |  | 2.3E-02 | | 3.3E-03 | |  | | | | |  | |
|  |  |  |  | |  | |  | | | | |  | |
| ***fix*-cluster and *ccoPQON* operon (aerobic respiration)** | | | | | | | | | | | | | |
| RPA0011 | orf | *rpa0011* | -0.72 | | 0.55 | |  | | | | |  | |
| RPA0012 | possible fixS | *rdxS* | -0.22 | | 0.75 | |  | | | | |  | |
| RPA0013 | putative cation (heavy metal) transporting ATPase | *rdxI* | -0.62 | | 0.55 | | 0.74 | | | | | 1.12 | |
| RPA0014 | orf | *rdxH* | -0.52 | | 0.75 | |  | | | | |  | |
| RPA0015 | 4Fe-4S ferredoxin, iron-sulfur binding domain | *fixG* | -0.52 | | 1.45 | |  | | | | |  | |
| RPA0016 | cytochrome-c oxidase fixP chain | *ccoP* | -0.42 | | 1.15 | | 0.84 | | | | | 1.32 | |
| RPA0017 | cytochrome oxidase subunit, small membrane protein | *ccoQ* | -0.52 | | 1.15 | |  | | | | |  | |
| RPA0018 | cytochrome-c oxidase fixO chain | *ccoO* | -0.62 | | 1.15 | | 0.84 | | | | | 1.22 | |
| RPA0019 | cytochrome-c oxidase fixN chain, heme and copper binding subunit | *ccoN* | -0.62 | | 0.55 | |  | | | | |  | |
| Average |  |  | -0.53 | | 0.89 | | 0.81 | | | | | 1.22 | |
| P-value |  |  | 4.6E-06 | | 4.2E-05 | | 7.0E-04 | | | | | 2.3E-03 | |
|  |  |  |  | |  | |  | | | | |  | |
| **pII gene cluster (ammonium and nitrogen regulation)** | | | | | | | | | | | | | |
| RPA0272 | GlnK, nitrogen regulatory protein P-II | *glnK1* | -0.42 | | -0.65 | | 0.04 | | | | | -0.08 | |
| RPA0273 | ammonium transporter AmtB / predicted SECONDARY transporter of H+/NH4+ | *amtB1* | -0.12 | | 0.35 | |  | | | | |  | |
| RPA0274 | GlnK, nitrogen regulatory protein P-II | *glnK2* | -0.52 | | 0.95 | |  | | | | |  | |
| RPA0275 | putative ammonium transporter AmtB / predicted SECONDARY transporter of H+/NH4+ | *amtB2* | -1.02 | | 1.05 | |  | | | | |  | |
